# BMWP: the first Bengali math word problems dataset for operation prediction and solving

**DOI:** 10.1007/s44163-025-00243-7

**Published:** 2025-03-13

**Authors:** Sanchita Mondal, Debnarayan Khatua, Sourav Mandal, Dilip K. Prasad, Arif Ahmed Sekh

**Affiliations:** 1 School of Medical Science and Technology, IIT Kharagpur, Kharagpur, West Bengal 721302 India; 2 Department of Mathematics and Statistics, Vignan’s Foundation for Science, Technology and Research, Guntur, Andhra Pradesh 512213 India; 3 School of Computer Science and Engineering, XIM University, Puri, Odisha 752050 India; 4 Department of Computer Science, Uit the Arctic University of Norway, Tromsø, 9017 Norway

**Keywords:** Bengali math word problems, Arithmetic operation prediction, Bengali NLP, Recurrent neural networks

## Abstract

Solving math word problems of varying complexities is one of the most challenging and exciting research questions in artificial intelligence (AI), particularly in natural language processing (NLP) and machine learning (ML). Foundational language models such as GPT must be evaluated for intelligence, and solving word problems is a key method for this assessment. These problems become especially difficult when presented in low-resource regional languages such as Bengali. Word problem solving integrates the cognitive domains of language processing, comprehension, and transformation into real-world solutions. During the past decade, advances in AI and machine learning have significantly progressed in addressing this complex issue. Although researchers worldwide have primarily utilized datasets in English and some in Chinese, there has been a lack of standard datasets for low-resource languages such as Bengali. In this pioneering study, we introduce the first Bengali Math Word Problem Benchmark Data Set (BMWP), comprising 8653 word problems. We detail the creation of this dataset and the benchmarking methods employed. Furthermore, we investigate operation prediction from Bengali word problems using state-of-the-art deep learning (DL) techniques. We implemented and compared various standard DL-based neural network architectures, achieving an accuracy of $92 \pm 2\%$. The data set and the code will be available at https://github.com/SanchitaMondal/BMWP.

## Introduction

**Fig. 1:**
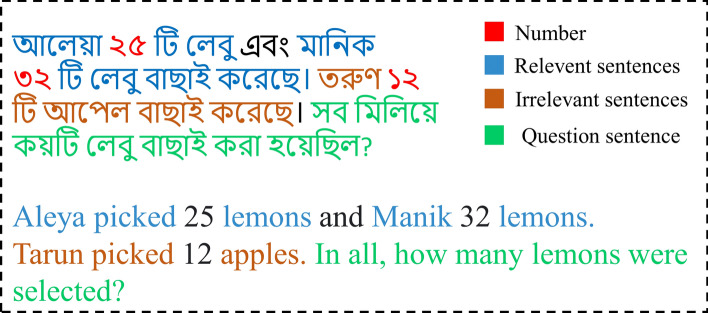
The figure illustrates the components of a Bengali math word problem. The problem is divided into four categories: problem statement, numeric values, contextual clues, and question target. These categories are highlighted in different colors to emphasize their roles in understanding and solving the word problem

One of the most challenging research problems in natural language is automatically solving math word problems; interpreting and resolving real problems with the texts is considered problematic: the AI’s skills to analyze language and reason present the most significant difficulties. It becomes more challenging to answer mathematical reasoning in regional languages. Although this research has been ongoing since the 1960s, it has recently accelerated due to the development of NLP, ML, DL, and Large Language Models (LLMs) [1] over the past eight years. Figure 1 shows how Bengali math word problems are structured. The problem statement provides context, numeric values offer the data required for calculations, contextual clues help establish relationships, and the question target defines the goal. This structured categorization highlights the unique challenges of interpreting word problems in Bengali, such as contextual ambiguity and linguistic complexity.

Among the resources offered by Mathematical text such as Word Problems (MWPs), the most uncommon yet interesting features are supposed to be the concept of linguistic understanding and mathematical reasoning. In this respect, MWPs provide the interpretation of natural language problem descriptions and identification of related values before deriving solutions using specific mathematical operations. MWPs are used more extensively in education to test the problem solving capacities of students and as benchmarks for AI models against tasks that add comprehension of the languages to reasoning. Even if there is considerable advancement in the creation of English MWP because of the availability of large datasets, regional languages such as Bengali come with a hefty price tag, regarding their linguistic complexity and lack of supporting resources. Thus, the study here presents a base for future research in the field by focusing on Bengali MWPs and offering a clean new dataset, the BMWP, to pursue research in this direction.

**Fig. 2:**
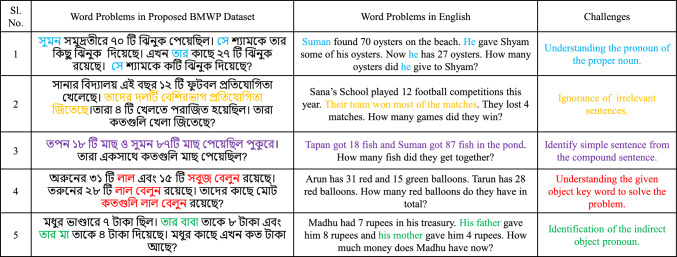
Some challenges of the Bengali word problems. The figure highlights specific challenges inherent in Bengali word problems, including pronoun resolution, identifying irrelevant sentences, distinguishing simple and compound sentences, and understanding object keywords. Each challenge is represented with distinct colors to illustrate their complexity

The current state-of-the-art systems use several large-scale datasets made available to solve math word problems in English and a few regional languages. Researchers have released the majority of annotated datasets of math word problems in recent years in English, such as Alg514 [2], which has 514 algebraic word problems, Dolphin18K^1^ which contains 18k annotated mathematical word problems, AllArith [3] containing 1492 arithmetic problems, MAWPS [4] (3320 arithmetic word problems), MathQA^2^ (37,000 mixed word problems with the rationale, options, and correct options for each), AQuA^3^(100,000 Algebraic questions answering with rationales, options, and correct answers), MATH^4^(12,500 competition word problems with step-by-step solutions), and so many others. Concerning other regional languages, Math23k^5^ [5] is a dataset of word problems with 23,162 different problems in Chinese. Recently,  Sharma et al. [6] proposed a Hindi word problem dataset with 2336 word problems. From the literature, we also see three very recent publications in Arabic [7], Turkish [8], and Korean [9]. Table 1 gives the details of the datasets in different languages with descriptions. However, there is no significant data set for the Bengali language, which is the seventh most spoken language in the world, Bengali. With 8653 math word problems in the Bengali language, we have proposed a data set called Bengali math word problems (BMWP). We have manually crafted Bengali word problems from Bengali textbooks of the second and third standards in elementary-level education. The word problems of our dataset consist of some challenges shown in Fig.  2. For example, pronouns must be understood in terms of their referents. However, this is not the case for compound sentences. The parsing task becomes more complicated when we look at these types of clauses. Important keywords also include recognizing object keywords like quantity and/or relationship valued in a problem. All these features help to construct a BMWP dataset. It also documents that the collected data are real problems faced by students between the ages. We have taken the problems from the proposed data set in the figure and identified the challenges by indicating the words.

### Challenges and research gaps

Here are some key research gaps in solving mathematical word problems, especially in regional languages like Bengali:

*Linguistic complexity:* Understanding contextual clues and solving pronouns in Bengali word problems is challenging due to linguistic uniqueness.

*Limited datasets:* There are fewer large-scale annotated datasets for Bengali compared to English, making it harder to train effective models. Ineffective translation of English into Bengali for such cases is ineffective.

*Integration of skills:* Combining linguistic understanding with mathematical reasoning is difficult, particularly in regional languages with complex syntax.

*Model adaptation:* Deep learning models trained in English data may not perform well in Bengali without significant adjustments and additional training data.

*Educational relevance:* Developing practical tools for students and educators in Bengali-speaking regions is essential to address real-world educational challenges.

These gaps highlight the need for more research and resources to improve AI’s ability to solve math word problems in regional languages.

### Training procedure

In our study, we trained the models using specific hyperparameters to ensure optimal performance. We set the number of epochs to 100, allowing the models sufficient time to learn from the data without overfitting. The learning rate was set at 0.001, which provided a balance between convergence speed and stability. We used a batch size of 16 to ensure efficient training while maintaining the ability to generalize well. Additionally, we used Adam Optimizer, known for its effectiveness in training deep learning models. These settings were chosen based on standard practices and previous research, ensuring a fair and consistent comparison between different models. By adhering to these configurations, we aimed to provide a reliable baseline to evaluate the effectiveness of each model in solving mathematical word problems in Bengali.

The primary contribution of this study is undoubtedly the production of a new dataset with second and third standard levels of arithmetic word problems in the Bengali language. The language is the seventh^6^ most spoken language in the world. We took great care to ensure that the word problems in the dataset included all four fundamental operations with various word problem types. Section contains the data collection and preparation specifics. The second clear contribution is the application of a deep learning (DL) approach in the prediction of operations leading to the creation of the final equation of a given word problem. To arrive at the answer, this equation was solved.

The rest of the article is organized as follows. Section 2 describes the previous work on mathematical word problems in English and other regional languages in this paper mainly by introducing a data set. In Sect. 3, we have delineated the construction and annotation of the proposed dataset. Next, Sect. 4 shows the proposed algorithms for solving the Bengali math word problems. The results of the experiment and the discussion are described in Sect. 5. In the end, Sect. 6 concludes the work.

## Related work

Over the past six decades, researchers in cognitive science, child psychology, problem solving, education, etc. have worked. have struggled to find an effective method to solve arithmetic math word problems (MWPs) [10–17]. Researchers in artificial intelligence occasionally focussed on it, attempting to find an autonomous solution based on computational representations. It was one of the first uses of computers, dating back to the 1960s [18, 19]. The solutions of word problems are discussed in detail in [20] and [21]. In recent years, much work has been done based on decades of research on MWPs [22].

Over the years, several large-scale datasets have been made available to solve mathematical word problems, including AQuA [23], which contains 100K complex MWPs, MathQA [24], which includes 37K word problems in English, and Ape210K [25], which contains 210K problems and 56K templates. In an earlier work [26, 27], statistical machine learning techniques were introduced to address MWP. Recently, more diversified datasets have come into focus instead of massive datasets. The difficulties of unbalanced lexical variety, difficulty level, and problem type distribution of MWPs, incorrectly annotated equations, and answers in large MWP datasets have been discussed by [28]. The challenge data set SVAMP was introduced [29], yet the best accuracy of cutting-edge solvers is substantially lower. The benchmark data sets are biased and contain word problems with significant lexical overlap, as demonstrated by [3].

Math word problem solving is an exciting research problem of AI and its subfields such as NLP, ML, and DL. It seems one of the finest research problems that addresses some of the critical issues of AI, like language understanding and representation, developing problem-solving reasoning and logic, goal-based systems and learning, etc. Although the research problem is ancient and was introduced by [9] in the 1960s. We have seen some development in the decade of the 1980s on tiny datasets of few-word problems. In the last decade, several attempts have been made to solve word problems using different techniques and approaches. With the advancement of machine learning and deep learning techniques, good system performance has been observed in recent years. However, most solved English Math word problems because the datasets were available. We mentioned some of the significant datasets in Table 1. In the literature, we find some of the remarkable research work that developed datasets and solved with good performance in recent times [3, 28–30]. Table 2 lists different methods and benchmarked results.

As our primary objective is to develop a dataset in Bengali and create a benchmark, we explain the datasets in languages other than English.

**Table 1:** Summary of major math word problem datasets across various languages, detailing their size, origin, and translation status. It highlights the lack of a comprehensive Bengali dataset until the creation of BMWP

Dataset	Description	GT	Language
Alg514 [2]	Corpus of algebric word problems	514	English
Dolphin18K [31]	Mathematical Word Problems	18,000	English
AllArith [3]	Arithmetic Word Problems	1492	English
MaWPS [4]	Arithmetic and Algebric Word Problems	3320	English
MathQA [24]	Multiple-choice Math Word Problem	37,000	English
AQuA [23]	Multiple-choice Algebric Word Problems with Natural Language Answers Rationales	100,000	English
MATH [32]	Mathematics Aptitude Test Problems	12,500	English
DRAW-1k [33]	General algebra word problems	1000	English
MWPs [34]	Math word problems	1078	English
HMWP [35]	arithmetic word problems, equations set problems, and non-linear equation problems	5491	English
Math23k [5]	One Unkowm Variable Linear Mathematical Word Problems	23,000	Chinese
ArMATH [36]	Single Variable Primary School Math Word Problems	6000	Arabic
MathQA [8]	Mathematical Word Problems from Probability, Physics, Geometry and Gain-Loss	37,200	Turkish
KoTAB [37]	Arithmatic Math Word Problems of Linear Equations with Single Variables	1162	Korean
HAWP [6]	Arithmatic Word Problem	2336	Hindi

**Table 2:** Summary of major math word problem datasets across various languages, detailing with the methods and accuracy

Dataset	Methods	Accuracy	Language
Alg514 [2]	Sequence-to-sequence model to map word problems to algebraic equations	70.3%	English
Dolphin18K [31]	Sequence-to-sequence model with attention mechanisms	79.7%	English
AllArith [3]	Latent structured SVM	77.19%	English
MaWPS [4]	Reducing lexical and template overlap of the data, and grammatically checker	–	English
MathQA [24]	Neural sequence-to-program model enhanced with automatic problem categorization	54.2%	English
AQuA [23]	Rationale generation approach	36.4%	English
MATH [32]	Autoregressive language models	40%	English
DRAW-1k [33]	Monte-Carlo algorithm	79.7%	English
MWPs [34]	A meaning-based approach, MeSys	81.5%	English
HMWP [35]	Universal Expression Tree (UET)	44.83%	English
Math23k [5]	Hybrid model (combination of RNN and similarity-based retrieval model)	47.7%	Chinese
ArMATH [36]	Transfer learning	74.15%	Arabic
MathQA [8]	Sequence-to-sequence neural model	72%	Turkish
KoTAB [37]	Modified TAB (Template-based Arithmetic Solver with BERT)	99.5%	Korean
HAWP [6]	BiLSTM	34.82%	Hindi

Very few datasets in other languages are available, as we stated earlier. In this paper, we describe some of the datasets in Chinese, Hindi, Arabic, and Korean. In Chinese, we have seen Math23K [5] datasets. HAWP (Hindi Arithmetic Word Problems), a dataset that includes 2336 Hindi arithmetic word problems, has been presented by Sharma et al. [38]. They have also created fundamental techniques for solving these word puzzles. They have suggested a brand-new evaluation method that takes equation equivalence into consideration for word problem solvers. KoTAB, a modified version of the TAB framework (BERT-based template arithmetic solver) used to handle English math word problems, is a Korean BERT-based template arithmetic solver that was proposed by Seo Ki et al. [37]. They produced CC_Ko and IL_Ko by translating the two English MWPS datasets, IL and CC [39]. All of the exercises for CC and IL were gathered from websites dedicated to online mathematics education. The four arithmetic operations $+,-,*,/$ are used in every problem in both datasets to solve a linear equation with a single variable. There are 600 problems in the CC dataset and 12 templates, while there are 562 problems and 12 templates in the IL dataset.

The first comprehensive dataset for Arabic MWPs, provided by Alghamdi et al. [36], contains 6,000 examples of elementary school arithmetic problems written in Modern Standard Arabic (MSA). Deep learning models are then used to construct and test Arabic MWP solvers using this dataset.

For the first time, Kim and Chun [40] have suggested a Korean data generator to overcome the lack of public Korean datasets. The problem category and data variation were included in the suggested data generator. It also contains 42 subclasses and 4 types of problems. Four categories make up the data variation, giving the model more sturdiness. During the experiment, a total of 210,311 bits of data, including 210,000 data points, were generated. There were 150,000 data points in the training dataset. With 30,000 data points each, the validation and test datasets. In addition, 311 issues were taken from commercially accessible books on math issues.

Turkish natural language processing (NLP) tasks that make use of neural models and pre-trained language models have been introduced by Gedik [8]. Furthermore, no Turkish data set has been created to apply neural models to MWP tasks. He has used a machine translation technique to convert the well-known English MWP datasets into Turkish because of the lack of data. He made manual corrections and created the corpus to add to the body of literature. The first dataset is created by integrating the elementary school math questions from the MAWPS, ASDiv-A, and SVAMP databases. By adding a few manual queries, 4164 MWP data are delivered in total. As the second dataset, 37,200 data points from the MathQA benchmark dataset were used. This dataset was chosen because it is among the most difficult datasets, has a reasonable volume of data, and covers a wide range of problems from many perspectives.

Several large-scale data sets have been produced over the years to address mathematical word problems, mainly in high-resource languages like English or Chinese. Notable examples are MathQA, AQuA, and Math23k, which have made substantial strides in natural language processing and machine learning techniques to address such problems. They, however, lack diversity concerning linguistic structures and do not recognize the intricacies of a low-resource language such as Bengali. The BMWP, on the other hand, fills this gap with special emphasis on the different linguistic, syntactic, and semantic problems in Bengali, such as very complex sentences, compounds like sandhi-viched, and references with pronouns that need the context to be correctly understood. Unlike many datasets, which are primarily translations or fabricated problems, 92% of BMWP is from real authentic Bengali textbooks, which makes it culturally and contextually appropriate. This gives BMWP value to represent real-world scenarios of Bengali-speaking students and advances to the next stage in NLP research in low-resource languages.

Table 3 shows the regional datasets for MWP. Note that most of the existing datasets are translated into English. We have collected the 92% mathematical word problems from native Bengali books used in a state-of-the-art education system and other 8% translated from English datasets.

**Table 3:** Here are some existing mathematical word problems in regional languages that are translated from English word problems

Dataset	Language	Samples	Translated from English
Sharma et al. [38]	Hindi	2336	Yes
Seo Ki et al. [37]	Korean	1162	Yes
Kim and Chun [40]	Korean	210,311	No
Gedik [8]	Turkish (Combined)	4164	Yes
Gedik [8]	Turkish (MathQA)	37200	Yes
Alghamdi et al. [36]	Arabic	6000	Yes
BMWP (Proposed)	Bengali	8653	Partially (8%)

Our proposed datasets are in Bengali and 92% collected from the native language. Other 8% of datasets are translated from English datasets

## BMWP dataset analysis

In this section, we propose an arithmetic word problem dataset (BMWP) in the Bengali language, with the aim of solving them. The seventh spoken language is ‘Bengali (or Bangla)’ out of all the languages spoken in the world. However, Bengali is one of the digitally low-resource languages in the research world. We have already discussed various math world problem datasets; however, we need a Bengali benchmark data set to understand the difficulties to solve. Due to its vast diversity of solving math word problems in regional languages, Bengali arithmetic word problem needs more consideration as it is much more challenging than English in terms of language complexity. We have assembled the BMWP dataset with four arithmetic operations. It includes 8653-word arithmetic problems in the Bengali language that deal with addition, subtraction, multiplication, and division. The dataset consists of word problems with a single equation with a single operation on characteristics. The dataset is publicly available at the link https://github.com/SanchitaMondal/BMWP.

### Data collection

Creating a comprehensive dataset for a low-resource language like Bengali is challenging, especially when there is a lack of available MWPs repositories. Although MWPs are an important component of the math curriculum in Bengali-medium schools, developing a diverse BMWP dataset is challenging. In this study, we have mainly focused on arithmetic word problems, which is drawn from elementary-level Bengali-medium textbooks used in schools across West Bengal (in India) and Bangladesh. The word problems are derived from textbooks from “ the West Bengal Board of Primary Education” and similar educational authorities in Bangladesh. These textbooks are aligned with the state and national curriculum, ensuring that the dataset reflects real-world classroom scenarios. Whereas, the problems are aligned with the cognitive abilities of elementary students from Grades 1 to 5 and are prioritized, ensuring simple single-step problems. To ensure diversity, we have included a mix of real-life scenario-based problems, such as shopping and school activities, alongside abstract mathematical problems. During the annotation process, we have manually organized this dataset into four attributes, such as “Problem”, “Equation”, “Solution”, and “Class”, each giving a distinct aspect in capturing the core elements of arithmetic word problems. The “Problem” attribute consists of Bengali text of MWPs from the aforementioned textbooks, whereas “Equation” includes mathematical formulation needed to solve each problem, the “Solution” consists of the final answer obtained by solving the corresponding equation and finally “Class” is added to categorize the problem based on the primary arithmetic operator(s), such as addition(+), subtraction(−), multiplication(*) and division(/) required for the solution. By structuring this, the BMWP dataset has ensured a balance between simplicity and comprehensiveness. It provides a solid foundation for various applications such as automated arithmetic problem solving, linguistic analysis, and the development of educational technology. The details of the dataset are as follows:

- Problems: 8653
- Equation Templates: 5 (As Table 4)
- Classes (or Operations): 4 [addition(+), subtraction(−), multiplication(*) and division(/)]

**Fig. 3:**
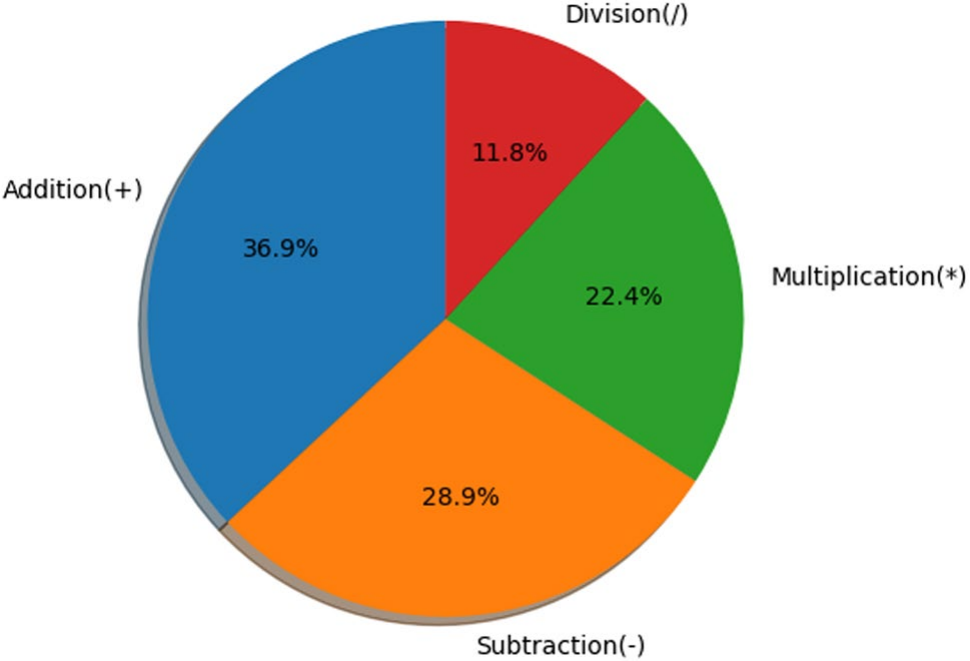
Distributions of the operations proposed in the dataset. We construct the dataset with 36.9% of addition, 28.9% of subtraction, 22.4% of multiplication, and 11.8% of division- type Bengali word problems

Figure 3 shows the volume of different operations in the dataset. In contrast, all segments indicate the percentage of arithmetic word problems. The BMWP is hand-crafted from Bengali-medium textbooks. We have collected 36.6% subtraction, 28.5% addition, 23.3% multiplication and 11.6% division word problems. The operation distribution in the figure reveals the cognitive focus of elementary textbooks where subtraction and addition are much more dominant than the other operations. This coverage of this distribution provides the dataset with a variety of operations in arithmetic and develops a good training set for modeling toward real-world Bengali math word problems. We manually annotated equations and arithmetic operations as class labels for word problems.

### Word problem annotation

In mathematics, arithmetic operations consist of addition, subtraction, multiplication, division, and modular arithmetic. In the proposed dataset, as we said, we consider four arithmetic operators: addition, subtraction, multiplication, and division as the output class label for training. We have accumulated all 8653-word problems and analyzed them to identify their operations toward solving the problem. We have gathered arithmetic word problems, which only include these basic four operations. The crafted data set is annotated with word problems with the desired equations and arithmetic operations of the problem. The dataset comprises 3197 addition, 2500 subtraction, 1936 multiplication, and 1020 division word problems. The grouping is shown as Table 5, which signifies the number of word problems for each arithmetic operation of the proposed dataset. Recent DL-based math word problem solvers are almost all built on the seq2seq approach, which first maps a problem into an equation called a template made up of numbers and operators. If the number is mentioned in the problem description, it is treated as a variable and changed to a placeholder based on where it is noted. Otherwise, it is a constant retained in the template as a number. For example, the problem template is shown in Table 4 as up to three quantities: N0, N1, N2. We manually crafted the templates for the word problems in the proposed dataset. We have the top five frequent templates in the BMWP dataset, as shown in Table 4.

**Table 4:** The list of the top five frequent equation templates in the BMWP dataset

Templates	Frequency
N0 + N1 (Addition of two items)	2568
N0 + N1 + N2 (Addition of three items)	546
N0 − N1 (Subtraction of two things)	2536
N0 * N1 (Multiplication of two things)	1918
N0 / N1 (Division of two things)	995

These templates represent common problem structures and are essential for training models to predict operations effectively

**Table 5:** Different operations in the BMWP dataset

Operations	#BMWP
Addition (Add multiple items)	3197
Subtraction (Subtract something)	2500
Multiplication (Multiply to get the answer)	1936
Division (Divide for getting the answer)	1020
Total	8653

The counting of the word problems corresponding to the four arithmetic operators in our dataset is given

## Building BMWP solver

**Fig. 4:**
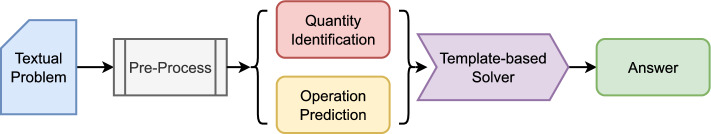
Flow of the proposed BMWP solver

The BMWP solver consists of four modules as shown in Fig. 4. The preprocessing step is used for data cleaning and tokenization. Next, quantity identification is used to identify different quantities associated with the question. The operation prediction module is used next to predict the operations. Finally, a template-based solver is used to predict the final answer.

In this section, we first describe the pre-processing of the BMWP dataset to solve the questions. Then we explain the two proposed approaches: the prediction of arithmetic operations and the solver approach for Bengali word problems.

### Data pre-processing

To prepare the Bengali Math Word Problems dataset for model training, we took help of the Bengali Natural Language Toolkit (BNLTK) [41]. For this case, tokenization had to be done because it helps to split text blocks into individual words or tokens, which will help to properly design the input features to be used by the neural network model.

This involved tasks like text cleaning; stripping off punctuation marks, extraneous spaces, and expression codes for a common representation of the data. The need to further segregate the information based on linguistic boundaries bestowed from the data: non-Bengali terms including English words were dropped to uphold corpus homogeneity. However, these measures were imperative for making the training set suitable for models since they prevented the contamination of data with irrelevant editorial flaws.

Contrary to most other work related to text, we avoided a textual processing stage with respect to removing all stop words and stemming them. Even simple boneheaded nearest-neighbor classifiers similar to those with full stop-word removal were found to be destructive when working with Bengali. This process had to be observed because Bengali does not have very simplified forms and some of these peculiarities are “compound words”, “conjunctive clause”, and “negativity” synonyms, where the former will not work when formulating solutions for math problems which are very challenging.

This line of action led to a very high level of data cleaning and specification of the dataset to be used in deep learning models, taking into account the language challenge as well as the computational nature of the problem.

**Algorithm 1:**
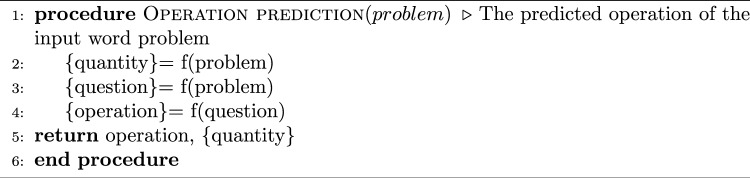
Arithmetic operation prediction

### Arithmetic operation prediction for BMWP

For the purpose of solving Bengali word problems, we have used the BMWP dataset to predict arithmetic operations. One of the major difficulties in NLP is solving arithmetic word problems in regional languages. In this method, the four fundamental operations are employed as the output class label for training and the Bengali math questions are used as the neural network’s input. We have used the recurrent neural network (RNN) methodologies SimpleRNN, GRU, BGRU, LSTM, Bi-LSTM, and Stacked LSTM. For building proposed neural network, In order to further classify data, we used three layers: an embedding layer with 100 dimensions as the input layer, hidden layers with predetermined hidden units followed by a dense layer, and an output layer, also known as a fully connected layer, which converts the output of the neural network layer into SoftMax probabilities for each class. We have used the GloVe word embedding model [42] for vector representation of word problems. Next, we have applied soft computing and cognitive-based approaches to identify operations. In this study, the loss function was assessed using categorical cross-entropy. Additionally, the Adam optimizer is used to train all the models in order to improve their performance across 40 epochs. Our approach for predicting the arithmetic operation of Bengali word problems is described in Algorithm 1. First, we use data preparation techniques to preprocess the Bengali word problem. Then, using the trained neural network, we determine the operation or the class label for the input problem and return the predicted operation. In order to finally answer the Bengali word problem, a mathematical expression is created using the extracted numerals (operands) and the operation (see below).

**Algorithm 2:**
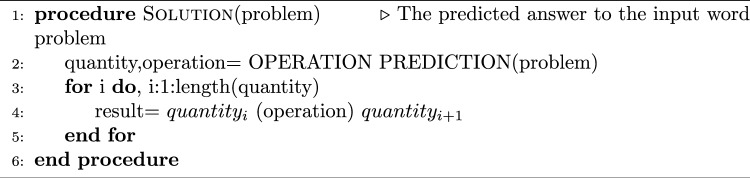
Arithmetic solution for classified BMWP dataset

### The solution approach for BMWP

Our method for solving the Bengali word problems in the dataset is described in Algorithm 2. Finding operands, or the numbers, from the input word problem is the first step in solving the Bengali word problem. After tokenizing the input word problems, we determine the numerals or operands for the word problems using steps 12–17 of Algorithm 2. Then, employing Algorithm 1, we determine the operation for the input problem. We can identify if a word problem is an addition, subtraction, multiplication, or division problem based on the projected operation, and we can obtain the solution or answer for the word problem. It is quite simple to build the final equation expression to be solved further and obtain the answer since we are able to locate the desired operation and operands for the Bengali word problem.

## Benchmarked results and discussion

The majority of word problem solvers are analyzed based on the accuracy of their equations or solutions. We have used the concept of operation equivalency of Bengali word problems with an accuracy of 88.5% on average. For the prediction model evaluation, we have analyzed mostly used classification metrics, such as precision, recall, and F1 score, with precision.

We used these as a metric to evaluate our model’s performance in solving mathematical word problems (MWPs) because it provides a clear and straightforward measure of effectiveness [21, 24]. Accuracy calculates the proportion of correctly solved problems out of the total attempted, making it easy to understand and interpret. This metric is particularly useful in educational settings, where the primary goal is to determine whether the model can consistently produce correct answers. By focusing on accuracy, we can directly assess the model’s ability to handle the mathematical and linguistic complexities of the problems, ensuring that it meets the fundamental requirement of generating correct solutions.

In our study on math word problem solving using AI, we selected LSTM [43], Bi-LSTM [44], Stacked LSTM [45], GRU [46], BGRU [47], SimpleRNN [48], and GPT-2 [48] to benchmark our proposed dataset due to their proven effectiveness in handling sequential data and capturing complex patterns in natural language processing tasks. LSTM (Long Short-Term Memory) networks are well known for their ability to remember long-term dependencies, making them suitable for understanding the context in word problems. Bi-LSTM (Bidirectional LSTM) enhances this capability by processing data in both forward and backward directions, providing a more comprehensive understanding of the sequence. Stacked LSTM, which involves multiple LSTM layers, allows capturing higher-level features and more complex patterns. GRU (Gated Recurrent Unit) and its bidirectional variant, BGRU, offer a more computationally efficient alternative to LSTM while maintaining similar performance, making them valuable for large datasets. SimpleRNN, though less complex, serves as a baseline to compare the performance of more advanced models. Finally, GPT-2, a transformer-based model, excels in generating coherent and contextually relevant text, making it a powerful tool for understanding and solving word problems. Using these diverse methods, we aim to comprehensively evaluate the performance and robustness of our dataset across different neural network architectures.

**Fig. 5:**
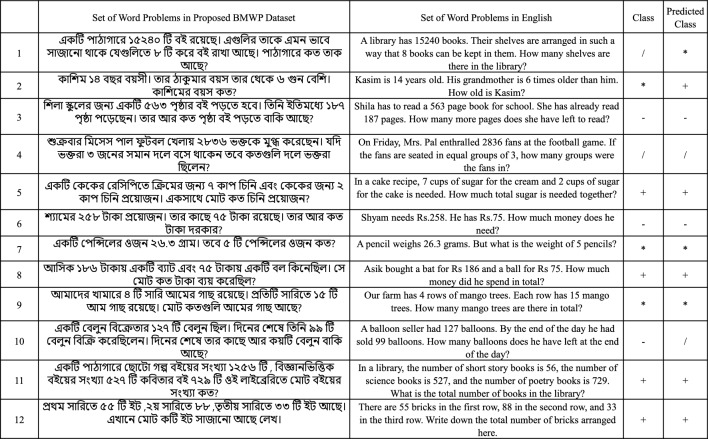
Examples of a set of word problems for evaluation on the prediction model. We have used the mentioned word problems to correspond with the arithmetic operators to validate the models. The predicted class is obtained from the best performing model, that is LSTM

We have taken examples of new word problems as defined in Fig. 5 for a case study. Despite all models, we got the best result for the LSTM model, the accuracy of the validation (30% of the data) is 90.42% with a precision of 95.62%, the recall of 90.24%, and the F1 score 92.85%. Table 6 compares the performance of all the models. In addition, we have fine-tuned the GPT-2 model for the operation classification. At first, the model with a classification head “GPT2ForSequenceClassification” is used, the GPT-2 tokenizer is used to preprocess input sequences and we have converted the labels into tensors. The dataset is divided into training and validation sets, and a custom collate function ensured dynamic padding during batch processing. During the training process, the model is fine-tuning for 10 epochs using the AdamW optimizer with a learning rate of 5e-5, and cross-entropy loss is used for the class prediction. The fine-tuned version of GPT-2 achieves an impressive accuracy of 88.2% on the 1729 test problems, far surpassing the baseline models. The evaluation process is carried out on TPU for efficient training, and the Hugging Face “transformers” library is used for this implementation.

**Table 6:** Performance evaluation on BMWP dataset

Models	Performance						
	Loss	Accuracy	Val Loss	Val Acc.	Precision	Recall	F1 score
LSTM [43]	0.2459	90.80%	0.3541	90.42%	95.62%	90.24%	**92.85%**
Bi-LSTM [44]	0.1427	93.35%	0.3686	90.42%	93.92%	90.41%	92.13%
Stacked LSTM [45]	0.1593	92.89%	0.4358	88.80%	91.90%	88.79%	90.32%
GRU [46]	0.1102	94.85%	0.4860	88.97%	91.84%	88.97%	90.38%
BGRU [47]	0.2116	91.98%	0.3389	89.67%	95.81%	89.66%	92.64%
SimpleRNN [48]	0.2606	91.32%	0.4672	87.01%	90.67%	87%	88.80%
GPT-2 [48]	0.2623	90.15%	0.4003	88.20%	86.55%	88.15%	87.22%

It is observed that the accuracy is highest as of 94.85%. 70% of the data are used for calculating the loss and accuracy, rest 30% of the data are used for calculating Val_Loss and Val_Accuracy. The precision, recall and F1_score are calculated on the whole dataset

### Challenges and failure cases

The assessment of foundational models such as GPT revolves around their ability to solve word problems in a zero-shot manner. Zero-shot learning refers to the model’s capability to solve problems it has never encountered during training, relying solely on its pre-existing knowledge. This is a critical measure of the intelligence and adaptability of a model. However, it has been observed that most pretrained models fail to answer these questions accurately, particularly when the problems are presented in low-resource languages such as Bengali.

To determine the precision of our approach, we compared the proposed work with ChatGPT, an AI large language model developed by OpenAI. ChatGPT is designed to understand and generate human-like text, making it potentially useful for solving mathematical word problems. However, it struggles with problems presented in Bengali due to the lack of training data in this language. We selected five Bengali word problems from the BMWP dataset and used ChatGPT to analyze them. The results showed that ChatGPT’s solutions were incorrect, highlighting its limitations in handling low-resource languages.

**Fig. 6:**
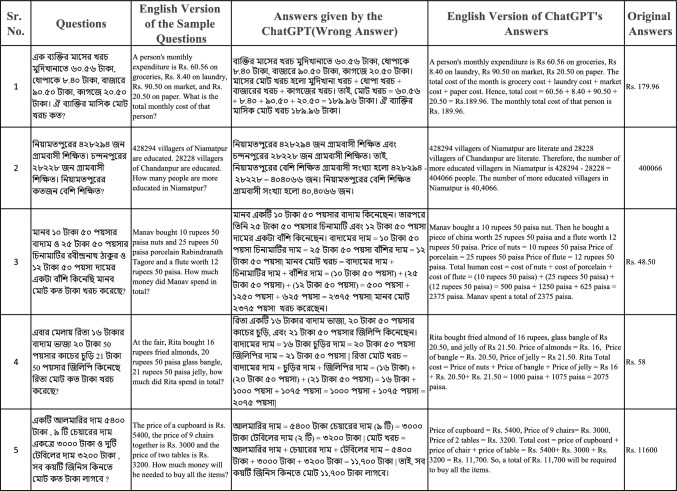
The figure compares answers provided by ChatGPT and our proposed model for five Bengali word problems. The figure highlights the inaccuracies of ChatGPT and the correctness of our model’s predictions, emphasizing the importance of specialized training for low-resource languages

In contrast, our proposed method provides precise responses for the group of word problems presented in Fig. 6. The figure shows that our model has a clear benefit over ChatGPT in solving Bengali math word problems. We go into some critical examples where the lack of training in the Bengali language for ChatGPT results in errors, such as representing pronouns or numeric values. This establishes the demand for such dedicated datasets and models for low-resource languages.

Additionally, we evaluated the BMWP dataset on several prominent large language models (LLMs), including GPT-2, mGPT-13B, mT5-Base, and Math_GPT2_sft. These models demonstrated significant limitations in the solution of the test problems, all of which achieved 0% accuracy. This indicates that despite their advanced capabilities, these models are not yet equipped to handle mathematical word problems in Bengali without additional training.

The failure of these models can be attributed to several factors. First, the linguistic complexity and syntactic structure of Bengali differ significantly from those of languages like English and Chinese, which are more commonly used in training datasets. Second, the mathematical terminology and problem-solving approaches in Bengali may not be well-represented in the training data, leading to poor performance. Finally, the zero-shot learning approach may not be sufficient for complex problem solving tasks that require a deep understanding of both language and mathematical concepts.

These findings highlight the need for more comprehensive and diverse training datasets, as well as the development of models specifically tailored to low-resource languages. By addressing these challenges, we can improve the accuracy and reliability of AI models in solving mathematical word problems across different languages and contexts.

## Conclusion

In this study, we introduce the first-ever dataset of mathematical word problems written in Bengali, named the Bengali Math Word Problem Benchmark (BMWP). This dataset is pioneering in its field, consisting of 8653 math word problems sourced from native educational books, rather than translations from English. The BMWP dataset focuses on the four basic arithmetic operations: addition, subtraction, multiplication, and division, providing a robust foundation for benchmarking.

To evaluate the quality and performance of our dataset, we conducted extensive research comparing it with other datasets used by deep learning (DL)-based models in the natural language processing (NLP) field. Our findings revealed that when trained on the BMWP dataset, the Long Short-Term Memory (LSTM) model significantly outperformed other models based on recurring neural networks (RNN) in terms of precision and accuracy. Specifically, the LSTM model achieved an impressive accuracy of 90.42% in the BMWP data set, highlighting its effectiveness in solving Bengali mathematical word problems.

However, our study also revealed several challenges and failure cases. The assessment of foundational models such as GPT revolves around their ability to solve word problems in a zero-shot manner. It has been observed that the majority of pre-trained models fail to answer these questions accurately, particularly when the problems are presented in low-resource languages like Bengali. For instance, when we tested ChatGPT on five Bengali word problems from the BMWP dataset, it failed to provide correct solutions, underscoring its limitations in handling low-resource languages.

Additionally, we evaluated the BMWP dataset on several prominent large language models (LLMs), including GPT-2, mGPT-13B, mT5-Base, and Math_GPT2_sft. These models demonstrated significant limitations in the solution of the test problems, all of which achieved 0% accuracy. This indicates that despite their advanced capabilities, these models are not yet equipped to handle mathematical word problems in Bengali without additional training. The linguistic complexity and syntactic structure of Bengali, along with the specific mathematical terminology used, pose significant challenges to these models.

While addressing challenges like linguistic complexity and pre-trained model limitations, future research should focus on expanding datasets, using transfer learning, and developing multilingual models using techniques such as cross-lingual embeddings and transformers (e.g., mBERT, XLM-R). Analyzing specific failure cases can highlight areas for improvement, such as refinement of model architectures and incorporation of advanced attention mechanisms. In addition, enhancing the explainability of solutions is crucial as it allows users to understand how models arrive at their answers, fostering trust and facilitating further refinement. By tackling these areas, we can advance the problem solving of multilingual mathematical words and make educational tools more accessible for low-resource language speakers.

Looking ahead, our goal is to expand the BMWP data set to include more complex mathematical problems, such as those involving differentiation, integration, and probability. This will enhance the dataset’s comprehensiveness and utility for a wider range of mathematical challenges. In addition, we plan to investigate advanced language models to further improve the understanding and solving of mathematical word problems. By addressing these challenges and failures, we hope to contribute to the development of more sophisticated AI models capable of handling diverse and complex mathematical tasks in low-resource languages like Bengali.

## Data Availability

Data is available in the link https://github.com/SanchitaMondal/BMWP.

## Footnotes

1 https://www.microsoft.com/en-us/research/project/sigmadolphin-2/

2 https://math-qa.github.io/

3 https://github.com/deepmind/AQuA

4 https://github.com/hendrycks/math/

5 https://ai.tencent.com/ailab/nlp/dialogue/#datasets

6 https://www.berlitz.com/blog/most-spoken-languages-world

## Code availability

Code is available in the link https://github.com/SanchitaMondal/BMWP.
